# Sirt1 is a tumor promoter in lung adenocarcinoma

**DOI:** 10.3892/ol.2014.2057

**Published:** 2014-04-10

**Authors:** XUE CHEN, DAISUKE HOKKA, YOSHIMASA MANIWA, CHIHO OHBAYASHI, TOMOO ITOH, YOSHITAKE HAYASHI

**Affiliations:** 1 Division of Molecular Medicine and Medical Genetics, Department of Pathology, Kobe University Graduate School of Medicine, Kobe, Hyogo 650-0017, Japan; 2 Division of Thoracic Surgery, Department of Surgery, Kobe University Graduate School of Medicine, Kobe, Hyogo 650-0017, Japan; 3 Department of Pathology, Nara Medical University, Kashihara, Nara 634-8522, Japan; 4 Department of Diagnostic Pathology, Kobe University Graduate School of Medicine, Kobe, Hyogo 650-0017, Japan

**Keywords:** sirtuin 1, lung adenocarcinoma, cellular senescence, apoptosis, hypoxia-inducible factor 1

## Abstract

Sirtuin 1 (Sirt1) is a nicotinamide adenine dinucleotide-dependent class III histone deacetylase. It reportedly can repress cellular apoptosis and senescence to affect DNA repair, stress response and aging. Notably, previous data have indicated that Sirt1 is both a tumor promoter and a tumor suppressor in tumorigenesis. However, Sirt1 expression in primary lung adenocarcinoma remains unknown. Immunohistochemical staining was performed to investigate Sirt1 expression in cancer cells in 125 consecutive resected cases of primary lung adenocarcinoma. Sirt1 expression was found to be increased in 26 (20.8%) of the 125 cases, which correlated significantly with five clinicopathological factors: Ki67 index, hypoxia-inducible factor 1 (HIF1) molecule expression, tumor-node-metastasis (TNM) classification, pulmonary vein invasion and lymphatic duct invasion. In the Sirt1-positive expression group, Sirt1 expression correlated with a higher Ki67 index and higher TNM classification, particularly for lymph node invasion and metastasis, and with a higher number of pulmonary vein invasion and lymphatic duct invasion. Additionally, a negative correlation was identified between HIF1-positive expression and Sirt1-negative expression. These results indicate that Sirt1 overexpression plays a promotional role in tumorigenesis and is closely associated with invasion and metastasis and, thus, it may be associated with prognosis.

## Introduction

Cellular senescence and apoptosis are potent tumor suppression mechanisms for the regulation of cell growth arrest and limitation of aberrant cell proliferation. It has been reported that age is the major risk factor for the development of cancer in mammals, and aging is often associated with the incidence of cancer, including human primary lung adenocarcinoma (1–3). Silent information regulator 2 (Sir2) has been reported to extend the lifespan by ≤70% in budding yeast. Mammals possess seven homologs of yeast Sir2, which are known as the Sirtuin family (Sirtuin 1–7). Sirtuin 1 (Sirt1) is the most thoroughly studied and is similar to yeast Sir2, which is a nicotinamide adenine dinucleotide-dependent class III histone deacetylase. The foremost function of Sirt1 is to deacetylate histone proteins, including H1, H3 and H4, and non-histone proteins. Non-histone proteins comprise of three major groups: Transcription factors, including p53, p73, androgen receptor, forkhead box protein O (FOXO), E2F1, hypermethylated in cancer 1 (HIC1) and nuclear factor-κB; signaling factors, including mothers against decapentaplegic homolog 7 and endothelial nitric oxide synthase; and DNA repair proteins, such as Ku-70. Sirt1, with its wide distribution in the cell nucleus and cytoplasm, has been found to regulate a series of normal physiological processes, including cell senescence, DNA repair and stress response (4–7). There are vast numbers of downstream molecules of Sirt1, including p53, FOXO1, FOXO3, FOXO4 and E2F1, which are regulated by Sirt1. Simultaneously, Sirt1 activity is regulated by its upstream molecules, for instance, p53, HIC1, E2F1, deleted in breast cancer 1 (DBC1), human antigen R (HuR) and active regulator of Sirt1 (AROS). Notably, a few downstream molecules of Sirt1, including E1F2 and p53, are also considered to be upstream molecules. These findings indicate that the interaction between Sirt1 and its upstream or downstream molecules is the reason for the multiple functions of Sirt1, and plays an essential and complicated role in cells.

Under normal conditions, the tumor suppressor gene HIC1 can negatively regulate Sirt1 transcription to inhibit Sirt1 expression. Additionally, it has been identified that in cell and animal models, there is a circular regulator loop among HIC1, Sirt1 and p53, in which HIC1 directly represses Sirt1 transcription, Sirt1 represses p53 activity by its deacetylation and inactivated p53 leads to HIC1 inactivation. However, a study by Tseng *et al* (8) indicated that this loop deregulates in patients with lung squamous carcinoma and lung adenocarcinoma (9). Similarly, DBC1 binds Sirt1 to form a stable complex to suppress the level of Sirt1, thus inducing cell apoptosis in response to oxidase (10).

Of course, certain agents can activate Sirt1 activity, including the tumor suppressor HuR and AROS (11). Knockdown of AROS can repress Sirt1 levels and enhance P21WAF1 to increase the G0/G1 population and cell apoptosis (12) Certain observations have indicated that the depletion of Sirt1 by small interfering RNA (siRNA) induces tumor cell death with no toxicity on normal cells (13). A previous study has provided strong evidence that Sirt1 is significantly overexpressed to function as a tumor promoter in mouse and human prostate cancer (14) and acute myeloid leukemia (15). However, previous studies indicate that Sirt1 is an inhibitor in colon cancer (16,17).

Certain study results indicate that regulated transcription of Sirt1 levels in cancer cells can affect the expression level of Sirt1. At least two feedback loops, Sirt1-p53 and Sirt1-E2F1, regulate this Sirt1 transcription. Two binding sites of p53 interact on the Sirt1 gene promoter and usually suppress Sirt1 gene transcription (6). In turn, Sirt1 can inhibit the activity of p53 by deacetylating its C-terminal Lys382 residue, thus further suppressing p53-mediated cell apoptosis following DNA damage. Acetylation of p53 is an indispensable process for the suppression of cell apoptosis (18). Thus, there is a Sirt1-p53 negative-feedback loop regulating the transcription of Sirt1. The other negative-feedback loop is located between Sirt1 and E2F1. Etoposide-mediated DNA damage induces E2F1 expression. E2F1 induces Sirt1 expression and is an apoptosis gene activator that can induce cell apoptosis dependently or independently of the p53 mechanism. E2F1 is also a downstream molecule of Sirt1, which deacetylates E2F1-induced transcription of target genes, including Sirt1, itself to prevent cell apoptosis (19).

In addition, it has been reported that the FOXO1, FOXO3 and FOXO4 members of the Forkhead transcription factor family interact with Sirt1, leading to increased resistance to stress and apoptosis, and thus to cancer cell survival (20–23).

According to previous studies into the biological properties of Sirt1, it was found to play an essential and complex role in normal physiological functions, and it was demonstrated that the dual function of Sirt1 caused by its upstream and downstream molecules was distributed differently in various tissues. However, it remains unknown whether Sirt1 is expressed in patients with primary lung carcinoma. In the present study, the expression of Sirt1 was analyzed immunohistochemically in surgically resected human primary pulmonary adenocarcinoma tissues from 125 patients. The effect of Sirt1 expression in tumor tissues on the outcome of these patients was also investigated.

## Materials and methods

### Patients

The data was analyzed for 125 patients (71 males and 54 females) who underwent surgery for primary lung adenocarcinoma subsequent to being diagnosed and treated at Kobe University Hospital, Japan, between 2001 and 2004. The study was approved by the Regional Ethics Committee for Clinical Research of Kobe University and conducted according to the principles of the Declaration of Helsinki. Dated and written informed consent was obtained from all patients. Primary tumors and adjacent non-neoplastic lung tissues were obtained at the time of surgery. Peripheral parts of the resected lung carcinomas were sectioned, evaluated by a pathologist and used for immunohistochemistry (IHC). All patients were enrolled consecutively. Detailed clinical and demographical information, prognostic factors and disease progression were collected retrospectively.

### IHC

Formalin-fixed paraffin-embedded specimens were sectioned at the maximal area of tumor mass into 5-μm-thick slices and sections were deparaffinized in xylene and rehydrated in ethanol, heat-treated for 20 min in Dako REAL™ Target Retrieval solution (no. S1699; Dako, Glostrup, Denmark) for antigen retrieval and 10 min in Dako Protein Block Serum-Free solution (code no. X909; Dako, Carpinteria, CA, USA). Rabbit anti-human Sirt1 monoclonal antibody ab32441 (concentration 1:200; Abcam, Cambridge, UK) was used as the primary antibody for detection of Sirt1. The Dako LSAB2 System-horseradish peroxidase (HRP) (DAB) kit was used for endogenous peroxidase blocking, treatment with a secondary antibody against anti-rabbit antibody and the visualization of HRP. Hematoxylin staining was used as the counterstain. Images of immunohistochemically stained sections were captured by a camera mounted on a Keyence BZ-8000 digital microscope (Keyence, Osaka, Japan).

### Classification of immunohistochemically stained patterns

Immunohistochemically stained sections were classified by means of light microscopy. For the assessment of the protein expression of Sirt1, samples were classified as Sirt1-positive when the ratio of stained cells in all epithelial cancer cells of a tumor tissue was >50%, and as Sirt1-negative if it was <50%. The cut-off value was set at 50%, as this value was statistically useful for this study (24).

The Ki67 index (Ki67 expression ratio), hypoxia-inducible factor 1 (HIF1) expression and tumor protein p53 expression were determined by the Division of Diagnostic Pathology, Kobe Medical University (Kobe, Japan). IHC was previously performed for 10 cancer-related proteins (including CDC45, HIF1, psf3, E-cadhelin and necl5) with the same paraffin-embedded specimens of the 125 cases investigated in the present study. The correlation between their expression in cancer cells compared with Sirt1 expression was also examined, however, only HIF1 showed a statistically significant different (P<0.05) (24–26).

### Statistical analysis

All statistical analyses were carried out with PASW Advanced Statistics 18 software (SPSS, Inc., Chicago, IL, USA). Baseline characteristics were expressed as percentages for categorical variables and as means ± standard deviation for continuous variables. Cross tabulation and χ^2^ tests were used to examine the association between Sirt1 expression and various clinicopathological parameters.

## Results

### Sirt1 expression in cancer cells of human lung adenocarcinoma

**Figure 1:**
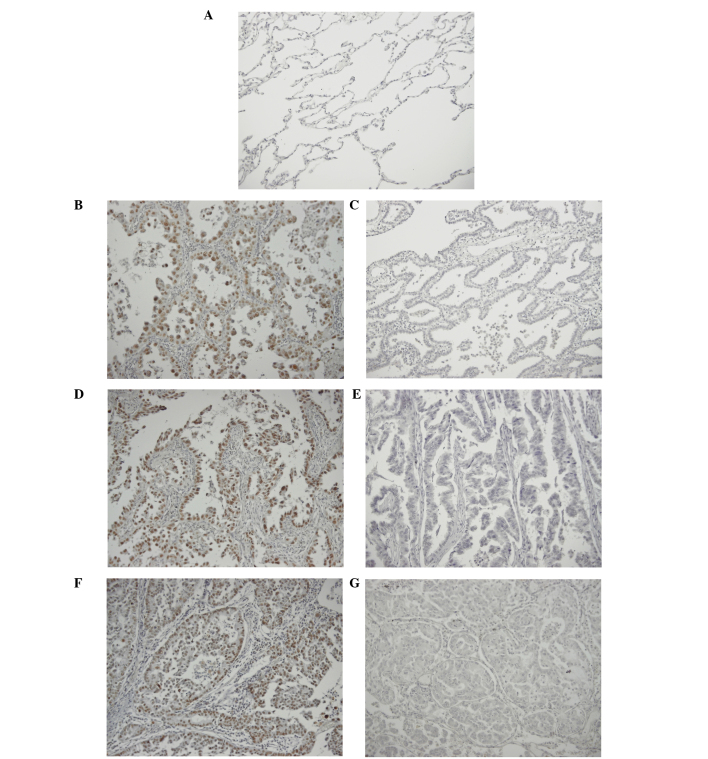
An immunohistochemical analysis of the Sirtuin 1 (Sirt1) expression status in cancer cells of human primary lung adenocarcinoma is illustrated. (A) Sirt1-negative expression in normal lung tissue. Sirt1-positive and -negative expression in (B and C) the lepidic predominant type of lung adenocarcinoma, respectively (magnification, ×200), (D and E) the papillary predominant type of lung adenocarcinoma, respectively (magnification, ×200), and (F and G) the acinar predominant type of lung adenocarcinoma, respectively. Cancer cells are stained brown.

The expression status of Sirt1 was determined in 125 lung adenocarcinoma and adjacent normal lung tissues by IHC, with the use of rabbit anti-human monoclonal antibody. In normal lung tissue, Sirt1 expression was not detected (Fig. 1A). In certain tumor tissues, the cancer cells were stained in a scattered pattern, and the ratio of the Sirt1-positive cells in such tissue was <10%. By contrast, some tumor tissues showed Sirt1-positive stained cells clustered in certain areas of the tissue, and the ratio of stained cells in such tissue samples was >80%. These tissue samples showing clustered staining were classified as Sirt1-positive (Fig. 1B, D and F; negative controls Fig. 1C, E and G). Thus, the status of Sirt1 expression was determined as follow: If >50% of cancer cells in any microscopic field (magnification, ×200) of tumor tissue showed staining, the tissue was considered Sirt1-positive; if the ratio of positive staining was <50% for all the examined microscopic field, the tissue was deemed Sirt1-negative. Of the specimens examined, 26 (20.8%) were positive for Sirt1 and 99 (79.2%) were negative for Sirt1 expression.

### Association between Sirt1 expression and clinicopathological characteristics of patients

**Figure 2:**
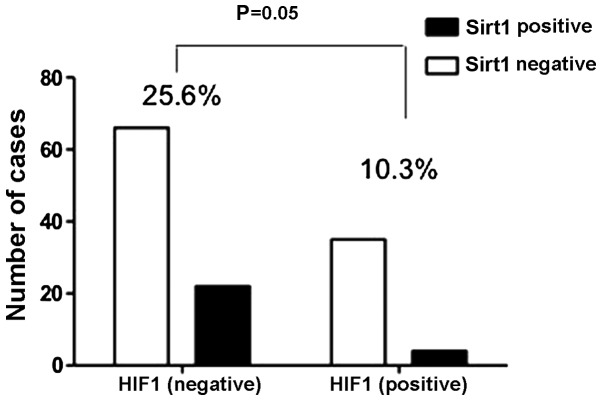
Hypoxia-inducible factor 1 (HIF1)-negative expression is associated with Sirtuin 1 (Sirt1)-positive expression. The P-value was determined using the χ^2^ test.

**Figure 3:**
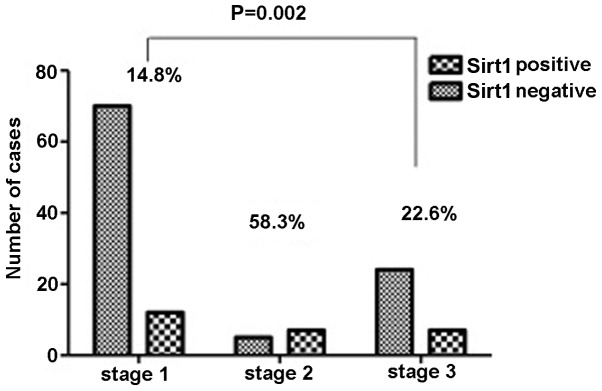
High tumor-node-metastasis (TNM) stage of the lung adenocarcinoma is associated with Sirtuin 1 (Sirt1)-positive expression. The P-value was determined using the χ^2^ test.

**Figure 4:**
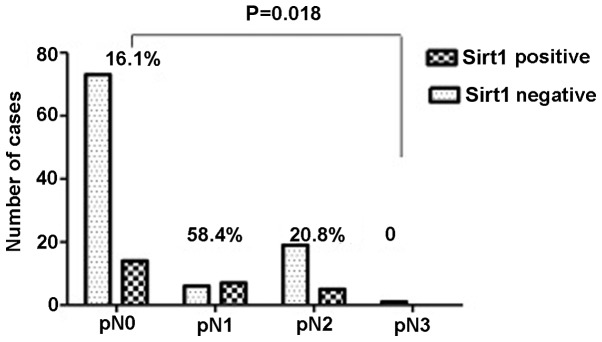
Lymph node invasion (pN) is associated with Sirtuin 1 (Sirt1)-positive expression. The P-value was determined using the χ^2^ test.

**Figure 5:**
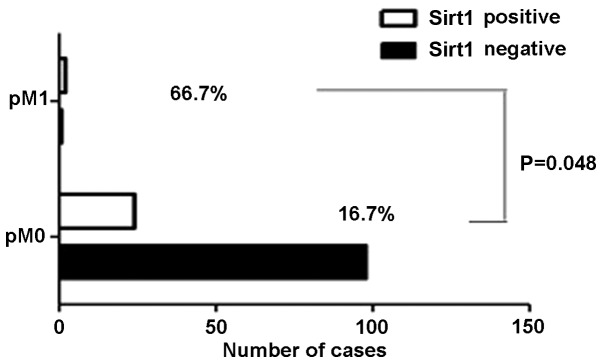
Metastasis (pM) is associated with Sirtuin 1 (Sirt1)-positive expression. The P-value was determined using the χ^2^ test.

**Figure 6:**
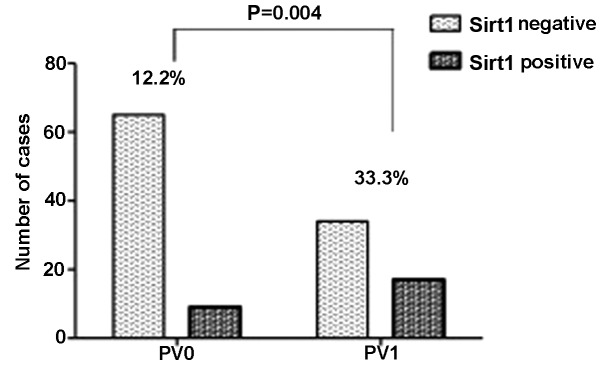
Pulmonary vein (PV) invasion is associated with Sirtuin 1 (Sirt1)-positive expression. The P-value was determined using the χ^2^ test.

**Figure 7:**
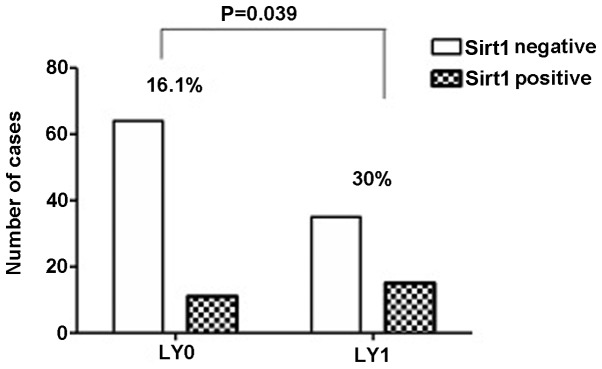
Lymphatic duct (LY) invasion is associated with Sirtuin 1 (Sirt1)-positive expression. The P-value was determined using the χ^2^ test.

In order to evaluate the role of Sirt1 in lung adenocarcinoma, Sirt1 expression was investigated in association with any of the clinicopathological variables in the 125 enrolled cases of primary lung adenocarcinoma (Table I). The results of the analysis revealed that Sirt1 expression was significantly associated with the Ki67 index (P=0.002), HIF1 expression (P=0.05) (Fig. 2), tumour-node-metastasis (TNM) stage (P=0.002) (Fig. 3), particularly in lymph node invasion (Fig. 4) and metastasis (Fig. 5), and with a higher number of pulmonary vein invasion (P=0.039) (Fig. 6) and lymphatic duct invasion (P=0.004) (Fig. 7). Sirt1 overexpression was not significantly correlated with age (P=0.617), gender (P=0.60), T factor (P=0.442), cancer invasion to the pulmonary artery (P=0.261) or p53 expression (P=0.577).

### Association between HIF1 and Sirt1 expression

HIF1 is a member of the HIF family that function as regulators and increase when cells become hypoxic due to oxidative stress (27). A significant association was found between HIF1 expression levels and a Sirt1-positive signal in patients with primary lung adenocarcinoma (P=0.05) (Fig. 2), and there was a negative regulation between them. A high level of expression of HIF1 indicates that cancer cells are in a hypoxic state. However, Sirt1 can regulate oxidative stress through indirect deactylation of FOXO3, eventually reducing the oxidative stress burden and thus leading to cell survival. Therefore, when cancer cells are suited to the surrounding environment through Sirt1 regulation, the molecular HIF1 level will decrease, resulting in a negative correlation between Sirt1 and HIF1 expression.

### Association of Ki67 index, TNM classification and tumor invasion with Sirt1 expression

In the present study, an extremely close association was found between the Ki67 index (determined by pathologists in the Division of Diagnostic Pathology of Kobe University Hospital) and Sirt1-positive expression (P=0.002). The Ki67 index frequently indicates cancer proliferation and has a strong correlation with clinical outcomes. This finding is consistent with the function of Sirt1, which is associated with cell survival and proliferation. In addition, it was found that the overexpression of Sirt1 is associated with a high TNM classification (P=0.002) (Fig. 3), particularly in lymph node invasion (P=0.018) (Fig. 4) and metastasis (P=0.048) (Fig. 5), but not primary tumor (P=0.442) and tumor size (P=0.151). Overexpression of Sirt1 also showed a significant association with pulmonary vein invasion (P=0.004) and lymphatic duct invasion (P=0.039) (Figs. 6 and 7), but without the pulmonary arteries (P=0.261). From these data, it was concluded that overexpression of Sirt1 is closely associated with invasion and metastasis.

## Discussion

Previous studies have indicated that Sirt1 is considered to play the part of a tumor promoter and tumor suppressor in tumorigenesis. These seemingly contradictory roles show that Sirt1 has a complicated function in tumorigenesis. The function of Sirt1 depends on the temporal and special distribution of its various upstream regulators and downstream targets in different tissue contexts (7). While it is recognized that tumor promoters and suppressors are significant in tumor development, no analyses of Sirt1 overexpression in a large number of surgically resected human cancer tissues have been reported, nor has the clinical significance of Sirt1 been properly ascertained.

In total, 125 surgically resected lung adenocarcinoma specimens were examined to determine the Sirt1 status in cancer cells and tissue clinically by IHC staining. The findings presented in the current study show for the first time that Sirt1 overexpression in lung adenocarcinoma tissue specimens is associated with HIF1 expression, Ki67 index, TNM stage, pulmonary vein invasion and lymphatic duct invasion.

A significant correlation was not identified between p53 and Sirt1-positive expression in the present study. Sirt1 was originally considered to be a tumor promoter, as it directly represses p53-mediated cell apoptosis and, as there is a negative-feedback loop between Sirt1 and p53, they regulate and interact with each other (5). However, in the current study, no significant association was found between Sirt1 and p53 (P=0.977). Similarly, a study by Jung-Hynes and Ahmad (4) demonstrated that Sirt1 overexpression occurs in both PC_3_ cells (which lack p53) and PC_3_-p53 cells (with wild-type p53), regardless of p53 in prostate cancer cells. In addition, a study by Kim *et al* (10) indicated that repression of Sirt1 activity by its specific inhibitor sirtinol or siRNA in MCF-7 cell leads to cell senescent-like growth arrest, indicating that the function of Sirt1 is disrupted by its inhibitor. Notably, Tseng *et al* (8) reported that the p53 mutation and p53 overexpression do not frequently occur in lung adenocarcinoma. Therefore, it is indicated that Sirt1 expression does not correlate with p53 in patients with primary lung adenocarcinoma.

However, in the present study, a direct significant association was not found between Sirt1-positive expression and the prognosis for patients with lung adenocarcinoma (P=0.238). Of the 125 cases enrolled in the present study, 32 were diagnosed with stage 3 of the TNM classification, but only three cases showed metastasis and succumbed, and of these cases, two showed Sirt1-positive expression (66.7%). It can therefore be considered that as the number of patients with stage 3 or metastasis of lung adenocarcinoma increases, overexpression of Sirt1 and the survival rate will be inevitably linked. It has been reported that the high level of HIF1 expression is associated with a good prognosis for lung cancer. HIF1 levels frequently increase in the extremely early stages of tumor progression, and are expressed *in situ* carcinomas and premalignant tissues. The function of HIF1 may be to decrease cell hypoxia and induce cell apoptosis (28,29). By contrast, one of the functions of Sirt1 is to enhance the chance of cell survival, with excellent growth and proliferation under hypoxic conditions. In the present study, a negative regulation between HIF1 expression and Sirt1-positive expression was found. Additionally, cells located in oxygen-poor conditions are more frequently observed in medium-term and advanced stages of cancer. These adaptable cells adjust to hypoxia and survive through Sirt1 regulation and possibly have a more aggressive phenotype and reduced sensitivity to anticancer treatment (29). Therefore, we suggest that Sirt1 participates in the initiation of tumors and furthermore its expression is more closely associated with medium-term and advanced stages of cancer and tumor development. Sirt1 may thus be associated with a poor prognosis for lung adenocarcinoma. In addition, hypoxia and oxidative stress can cause DNA damage. Sirt1 also plays a positive role in repairing double-strand DNA breaks. Erroneous DNA replication or repair causes unceasing proliferation of aberrant cells as a function of Sirt1 and the probability is high for these cells to become tumorous (30). DNA damage also accumulates with age and DNA repair defects can cause phenotypes resembling premature aging, thus prolonging the presence of senescent cells in a neoplastic microenvironment, which in turn may promote malignant progression of adjacent epithelia cells. In young organisms, cellular senescence can be considered an advantageous mechanism for reducing aberrant mutations or impeding exposure to oxidative stress, but it may be harmful in that it may promote phenotypes associated with old age and thus potentially contribute to tumorigenesis (31). With an increase in age, specific inhibitors of Sirt1 function, including HIC1 and DBC1, become weaker. They repress Sirt1 expression in normal cells, but with aging of the cells they may gradually lose their function to promote tumorigenesis (31).

Ki67 index and TNM classification are also significant indicators for clinical tumor development. The high Ki67 index and TNM classification indicate that cancer cells have a faster growth and differentiation in tumorigenesis. Furthermore, there is a strong possibility for invasion of the surrounding tissue and metastasis to other areas. These usually occur in malignant tumors and are associated with a poor prognosis for patients with lung adenocarcinoma. In the present study, it was found that a high Ki67 index and TNM classification are closely correlated with Sirt1-positive expression. Particularly, in TNM classification, Sirt1-positive expression is more closely associated with lymph node invasion and metastasis, but not with tumor size. Thus it can be observed that overexpression of Sirt1 is closely associated with invasion and metastasis. It is indicated that overexpression of Sirt1 may be correlated with a poor prognosis for lung adenocarcinoma again. These indicate that identifying an inhibitor based on the biological features of Sirt1 may make Sirt1 an ideal target for the development of potent anticancer drugs.

## Figures and Tables

**Table I:** Associations between the increased expression of Sirt1 and clinicopathological characteristics of 125 patients with lung adenocarcinoma.

Variable	Total	Sirt1-positive	Sirt1-negative	P-value
No. of patients, n (%)	125 (100)	26 (20.8)	99 (79.2)	NA
Age, years	85±8.685	70.46±4.623	66.83±9.307	0.617
Mean ± SD (range)	(42–84)	(60–79)	(42–84)	NA
Gender, n (M/F)	71/54	19/7	52/47	0.600
Succumbed/remained, n	30/95	8/18	22/77	0.364
T factor, n				
TI/T2/T3/T4	68/43/4/10	12/9/1/4	56/34/3/6	0.442
N factor, n				
N0/N1/N2/N3	87/13/24/1	14/7/5/0	73/6/19/1	0.018^a^
M factor, n				
M0/M1	122/3	24/2	98/1	0.048^a^
TNM stage, n				
I/II/III/IV	82/12/31/0	12/7/7/0	70/5/24/0	0.002^a^
PA invasion, n				
Positive/negative	24/101	7/19	17/82	0.261
PV invasion, n				
Positive/negative	51/74	17/9	34/65	0.004^a^
LY invasion, n				
Positive/negative	50/75	15/11	35/64	0.039^a^
p53 expression, n				
Positive/negative	67/58	14/12	53/46	0.977
HIFI expression, n				
Positive/negative	39/86	4/22	35/64	0.050^a^
Ki67 index, n				
Positive/negative	111/14	26/0	85/14	0.002

a Significant P-value.

SD, standard deviation; M, male; F, female; T factor, tumor factor; N factor, node factor; M factor, metastatis factor; PA, pulmonary artery; PV, pulmonary vein; LY, lymphatic duct; HIF1, hypoxia-inducible factor 1; Sirt, sirtuin; NA, not applicable.
